# Trends in radiology and *experimental* research

**DOI:** 10.1186/s41747-017-0006-5

**Published:** 2017-06-29

**Authors:** Francesco Sardanelli

**Affiliations:** Department of Biomedical Sciences for Health, Università degli Studi di Milano and Unit of Radiology, IRCCS Policlinico San Donato, Via Morandi 30, 20097 San Donato Milanese, Milan Italy

## Abstract

*European Radiology Experimental*, the new journal launched by the European Society of Radiology, is placed in the context of three general and seven radiology-specific trends. After describing the impact of population aging, personalized/precision medicine, and information technology development, the article considers the following trends: the tension between subspecialties and the unity of the discipline; attention to patient safety; the challenge of reproducibility for quantitative imaging; standardized and structured reporting; search for higher levels of evidence in radiology (from diagnostic performance to patient outcome); the increasing relevance of interventional radiology; and continuous technological evolution. The new journal will publish not only studies on phantoms, cells, or animal models but also those describing development steps of imaging biomarkers or those exploring secondary end-points of large clinical trials. Moreover, consideration will be given to studies regarding: computer modelling and computer aided detection and diagnosis; contrast materials, tracers, and theranostics; advanced image analysis; optical, molecular, hybrid and fusion imaging; radiomics and radiogenomics; three-dimensional printing, information technology, image reconstruction and post-processing, big data analysis, teleradiology, clinical decision support systems; radiobiology; radioprotection; and physics in radiology. The journal aims to establish a forum for basic science, computer and information technology, radiology, and other medical subspecialties.

## Introduction

The decision of the European Society of Radiology (ESR) to launch *European Radiology Experimental* occurred in the context of relevant trends influencing the future of radiology. In changing times, to explore (*to experiment*) new ways, methods, and opportunities is important for spreading awareness of changes and guiding adaption. This article describes these trends and how *experimental* research in radiology (and this new journal) can play a role. “Today’s research is tomorrow’s practice” [1], and experimental research is a key factor for entering the future through the main gate.

**Fig. 1:**
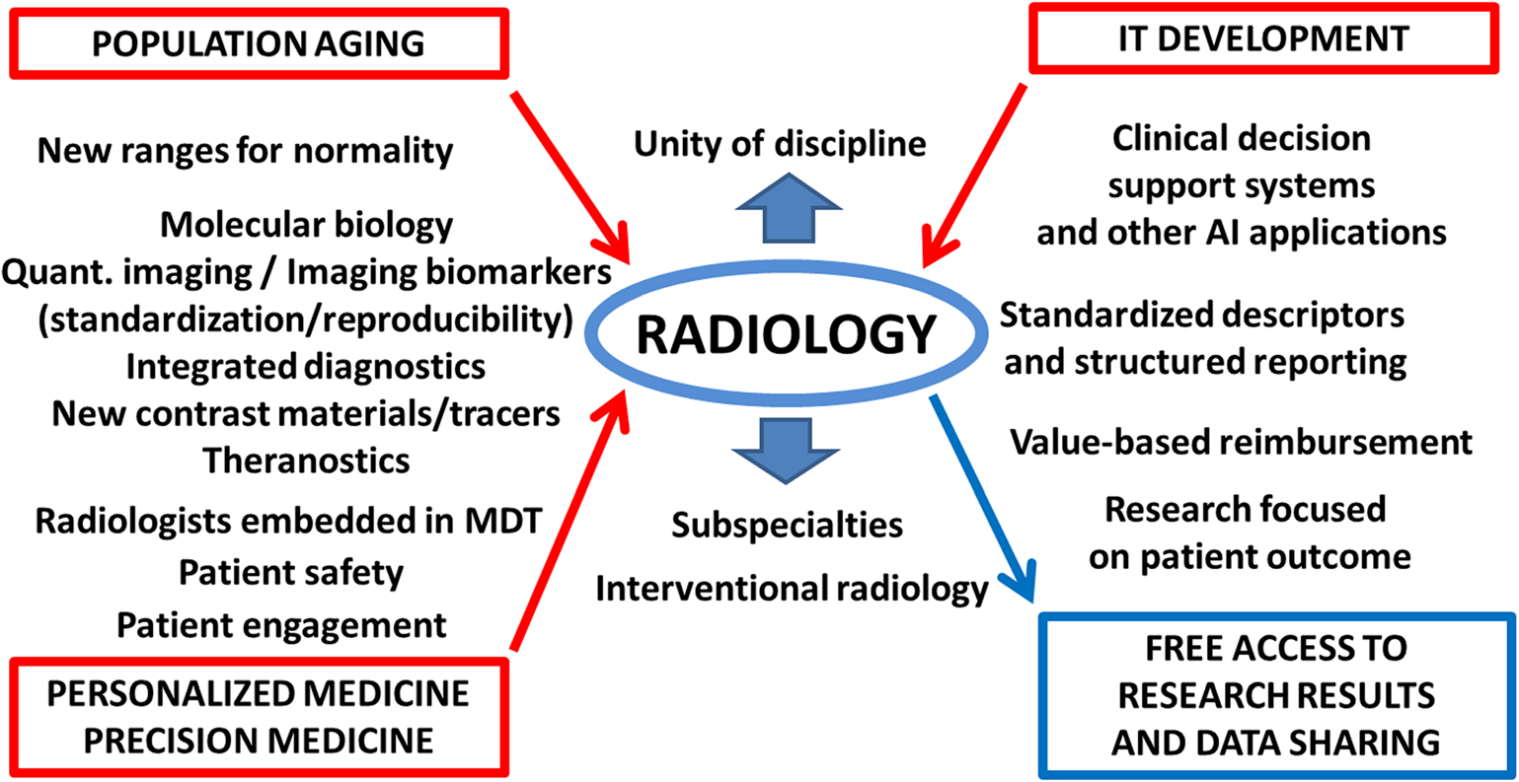
General and specific trends influencing radiology and radiological research. MDT, multidisciplinary team; IT, information technology; AI, artificial intelligence

Three general trends and seven radiology-specific trends will be outlined. Both general and specific trends interplay and many overlap. A graphical representation is given in Fig. 1, including relevant effects of the various trends. Then, different meanings of the word *experimental* and the structure and role of the new journal will be described.

## Three general trends

### Population aging

The first general trend is *population aging*, a major factor especially in Europe, North America, and China but also, although at a slower pace, in the rest of the world. In 2002, the United Nations defined population aging as *unprecedented, pervasive, enduring, and with profound implications for many facets of human life* [2]. Baby boomers are now aged. From 2010 to 2040, the population over 65 is expected to increase from 16.1 to 25.2% in more developed regions, and from 5.8 to 12.4% in less developed regions including China. The population over 80 will increase from 4.3 to 8.6%, and from 0.9 to 2.5%, respectively. In 2050, in more developed regions, one out of ten inhabitants will be over 80 [3].

Population aging has two major consequences. On the one side, physicians should extend their knowledge about *physiology of advanced elderly*, changing their paradigms about *normality*. To distinguish between normal and abnormal will become more challenging. What is the normal range of cardiac ejection fraction or the normal glomerular filtration rate of a 90-year-old *healthy* subject? What about *normal* size of subarachnoid spaces in the advanced elderly? Indeed, we need studies to generate new reference ranges, which also consider the increasing ethnic diversity due to migration, especially in Europe.

On the other side, aging determines epidemiologic trends. Ischemic and hypertensive heart disease, stroke, pulmonary infections, cancer, and diabetes still remain the most relevant causes of death in middle-upper and high income countries, with dementia being a major problem for healthcare systems in high income countries [4]. Image-based innovations in risk stratification, screening, clinical diagnosis, interventional therapy, and prognosis in these fields will be welcomed by the journal. Although risk stratification, screening, and prognosis seem to be outside the area of experimental research, this is not true. Image-based techniques and methods need tests, refinements, and specifications before they may be used in practice, as explained below when considering *quantitative radiology*. This is also the key for acquiring a deeper knowledge of the physiology of advanced elderly.

### Personalized and precision medicine

The second general trend includes *personalized medicine*, an approach which considers the individual characteristics of each subject for disease susceptibility, biology, and prognosis of diseases, and response to treatment. When the goal is to create a new taxonomy of human diseases based on *molecular biology*, we refer to the so-called *precision medicine* [5, 6]. This new *patient-centred* approach has profound implications for risk stratification and tailored screening or surveillance programs and personalized therapies. Molecular biology has a potential partnership with *molecular imaging*. As recently addressed by the ESR [7], *imaging biomarkers* can be used in all these steps, if our community is able to solve the challenge of standardization and reproducibility [8]. The relation between imaging features and genomics (*radiogenomics*) is adding more value to this patient-centred approach [9]. *Biobanks and imaging biobanks* will also play a role [10]. Potential exists for a future *integrated diagnostics* framework including both imaging and pathology, the latter of which has just become digital, as imaging is since many years.

Personalized and precision medicine does not end with the individual patient or patient’s disease. In oncology, *tumour heterogeneity* should be assumed as a major factor determining response to therapies [11]. A biopsy-based approach to multiple tumour lesions has practical limitations. Thus, radiology is well positioned for mapping cancer heterogeneity in the individual patient and guiding the *adaptive therapy*, especially if *molecular imaging*, *radiomics*, *radiogenomics* and *habitat imaging* techniques are used [6, 12, 13].

*Patient engagement* will improve personalized medicine, especially through the increasing use of mobile technology and the Internet. Automated emailing and interactive patient portals are already playing a role, offering the possibility of electronic access to medical records, images and reports [14]. Further research is expected on new software solutions for patient engagement in radiology, from scheduling procedures to follow-up examination reminders.

In the context of personalized and precision medicine, the research on *contrast materials* and *tracers* will play a big role. On the one hand, we should rethink individual dosages. One possibility is to consider the lean mass instead of the bodyweight for *dose tailoring* [15–17]. On the other hand, the development of new contrast materials and tracers will make the perspective for *personalized and precision medicine* more feasible. In this regard, *theranostics*, i.e. disease imaging and therapy together (targeted molecular imaging to follow the drug delivery pathway), is the most fascinating direction to follow [18].

### Information technology development

Information technology (IT) development is one of the most important trends impacting individual and social life worldwide. From current applications on mobile phones and tablets to applications of artificial intelligence (AI) to medicine, our future will be strongly influenced by IT. Radiology was the leading discipline in the medical digital era [6]. Thus, relevant IT and AI innovations are expected in radiology and we should try to keep a prominent position as IT innovators in the arena of medical specialties. To this aim, the cooperation with IT scientists is necessary.

Advanced techniques of quantitative imaging, AI applications to *clinical decision support systems*, and *big data analysis* are only a few examples of radiology-specific trends coming from IT development.

## Seven radiology-specific trends

### Subspecialties versus unity of discipline

The *tension between subspecialties and the unity of the discipline* is increasing. Radiology subspecialties have a long history, with neuroradiology being one major paradigm of full dedication [19]. Interventional radiology inclined to claim for becoming “a primary specialty with uniquely trained clinicians” [20] or “an almost autonomous clinical specialty” [21]. To have radiologists subspecialized in specific fields is an obligatory way to answer clinical needs, to maintain our central role in multidisciplinary teams, and last but not least, to guide both experimental and clinical research. The *radiologist embedded in a clinical team* seems to be the best model of radiology consultation, with a non-negligible trade-off paid to productivity [22]. Are we risking a fragmentation of radiology?

*To retain the unity of the discipline* is not an old conservative academic viewpoint but a current need, as shown by the following considerations: 1. General previous training can be an advantage in comparison to clinicians who practice imaging in their field (“neuroradiologists may offer a differential diagnosis that includes non-neurologic conditions based on their more general training” [19]); 2. Modern imaging techniques commonly explore the body of the patient also outside the area of interest (in the case of cardiac computed tomography, the detection of a lung tumour can be more important than coronary stenosis quantification [23]); moreover, in multidisciplinary cancer teams, radiologists are frequently asked for their opinion about diagnosis and interventional treatments of metastases all over the body; 3. The distinction among subspecialties is blurred, important fields are cross-bordered, and the identity of subspecialties is always changing and evolving while imaging techniques migrate from one subspecialty to another; 4. Organizational aspects favour a central radiology department due to the impossibility to have radiologists dedicated exclusively to one subspecialty; additionally, a central radiology department able to manage large and expensive equipment allows a more cost-effective work-flow; 5. Some hybrid systems require the combination of radiology and nuclear medicine expertise, suggesting a unified training program as has already been initiated under the unique “Radiology” denomination in 2014 in The Netherlands [24].

### Patient safety

A patient-centred approach also includes patient safety, in particular *radioprotection*, as highlighted by the European Society of Radiology (ESR) [25]. Radiologists should acknowledge the efforts made by the industry for a reduction of x-ray exposure, especially for computed tomography (CT). The possibility of performing coronary CT studies with much less than 1 mSv has been demonstrated [26, 27], while thoracic CT can be performed with less than 1 mSv (unenhanced studies) and less than 2 mSv (contrast-enhanced studies) [28].

These dramatic improvements imply two consequences. First, as suggested by the ESR [29], *renewal of radiological equipment* will be one of the most important factor driving the ionizing radiation exposure reduction in the next years. Second, *reporting of radiation dose*, if not already required by local regulations, will become a routine practice as indicated by the European Union Council Directive 2013/59 [30]. More preclinical (especially on phantoms) and clinical research is still expected on radioprotection. Research about the use of new hardware and software is welcomed by our journal. A similar reasoning applies for studies aimed at reducing the dose of both iodinated and gadolinium-based contrast materials, the latter especially after the so-called “brain deposition” issue [31].

### Quantitative imaging

Imaging procedures will provide more and more output, not only images but also clinical data, numbers, indices, the core of the so-called *quantitative imaging*. Digital images are intrinsically data [32]. In certain cases, data can be more important than images. Examples are bone mineral densitometry and trabecular bone score through dual energy x-ray absorptiometry, where reproducibility defines the smallest detectable difference and the time to follow-up [33–37]. This is crucial for *imaging biomarkers* to be used for radiomics and radiogenomics, in particular for MRI-derived parameters. Radiologists are generally not ready for this, being mainly trained for qualitative reporting. For a long time, quantitation has been limited to the use of electronic calipers for size or to region-of-interest-based measurements of tissue electronic density through Hounsfield units in CT.

We should not forget the counterintuitive evolution of imaging methods for evaluating the response to therapy of solid tumours, from the two-dimensional criteria (the cross-product) proposed by the *World Health Organization* in 1981 [38] to the one-dimension Response Evaluation Criteria in Solid Tumours (RECIST) 1.0 in 2000 [39], as well as the RECIST 1.1 simplification, including positron emission tomography only in 2009 [40]. Notably, the main requirement for any new parameter to be accepted by the non-radiological clinical world is *reproducibility*. This explains why a diameter is more reproducible than a cross-product and the slow adoption of volume measurement [41].

Without reaching reliable standardization and reproducibility, new imaging-derived parameters are deemed to remain research topics only. A lot of experimental work is needed on the path to an imaging biomarker development and acceptance, proving the concept, the mechanism, the principle, the efficacy and effectiveness up to its use as surrogate end-point in clinical studies [42].

### Standardized and structured reporting

Radiologic reporting is evolving towards standardized descriptors and diagnostic categories, in the context of *structured reports*. The Breast Imaging Reporting and Data Systems (BI-RADS), more than two decades after its first introduction in 1993 [43, 44], has been imitated in many other fields of diagnostic imaging [45–49] and the practice of radiology will follow this trend to facilitate the information transfer to patients and clinicians, including other radiologists.

### Search for a higher level of evidence

We will be increasingly asked to demonstrate that radiology works in favour of patients, not only in terms of diagnostic performance but also at higher levels of the evidence-based medicine hierarchy, which implies *impact on treatment, patient outcome, and societal effects* [50]. This is now practically evident in the paradigm shift from a *fee-for-service* to a *value-based* model for reimbursement [51], in the context of a reduction or at least an end of expansion of healthcare expenditure [52]. Even though this trend mainly implies large clinical studies, innovation in study design to show efficacy and effectiveness of radiological procedures can be proposed through our journal.

### Increasing relevance of interventional radiology

A major trend is surely the *increasing penetrance of interventional radiology*, which is a fundamental asset to improve the clinical profile of radiology [20, 21]. The role of minimally invasive image-guided therapies will expand in the next years, especially concerning interventional oncology. For the next generations it is of crucial importance that we continue to lead the way in device and method innovation in interventional radiology.

However, we should also try to build higher levels of evidence in favour of interventional radiology compared with standard methods, competing with other specialists working in the field [21]. Notably, the innovation of devices and methods is an easier task than building high-level evidence, as “most interventional radiologists lack expertise in the relatively challenging advanced methods used in comparative effectiveness and cost-effectiveness research” [20]. This challenge implies efforts in education and mentoring, beginning with training during post-graduation schools.

### Technological evolution

Last but not least, we have to consider the *continuous technological evolution of existing imaging methods*, the *introduction of new imaging methods* as well as *various effects of IT and AI development in the field of medical imaging*.

The general economic context will probably favour the evolution of existing imaging methods more than the introduction of new imaging methods. Imaging procedures will become faster and faster, as has already happened with CT and MRI, while new hybrid technologies will be proposed. An intriguing evolution of existing methods could be an *unprecedented portability of imaging devices* [6], especially in the emergency setting [53]. While phase-contrast x-ray may bring relevant innovations to radiodiagnostics [54–56], optical and photo-acoustic imaging may come to clinical practice [57, 58].

We should also take into consideration a sceptical view, such as that proposed by Eugen Lin in 2011, when he wrote [52]: “In many cases, our technology has reached a point where the marginal value of further advances for patient care may be minimal. […] I believe that there will be substantially fewer technologic advances implemented in routine clinical practice in the near future. But what of the much-touted molecular imaging? Although I do not doubt the potential of molecular imaging compared with existing anatomic imaging techniques, molecular imaging techniques will likely face the same substantial hurdles to reimbursement”. In any case, the initial evidence provided for new modalities or techniques in the experimental setting will be necessary for translation into clinical practice. *European Radiology Experimental* will provide a forum for this.

Among the technological evolution we can include *IT development*. Radiology information systems will be integrated in hospital and regional/national health information systems. All areas of innovation in this field and their interplays require research by radiologists and IT experts, as outlined by Nance et al. [59]: aggregation of electronic medical records, allowing radiologists to access clinical information (not only information provided by ordering clinicians) at hand when defining the protocol of a procedure or interpreting images, immediate use of clinical decision support systems for ordering, interpreting, and defining further patient management, internal peer review, tracking of resident training, communication of critical findings, quality control of technologists’ performances and communication between radiologists and technologists, customer service towards patients and referring physicians, surveillance and outcome measures, and data mining regarding previous issues (including radiation dose).

It is easy to predict that AI will be increasingly implemented in medical imaging systems. Examples of this trend are computer aided detection and diagnosis, advanced image analysis, such as *texture energy* and *deep learning* methods [6], fusion imaging, three-dimensional printing, structured reporting and new models of relations of radiologists with patients and referring physicians, teleradiology.

In particular, *clinical decision support systems* will be used by radiologists (management of additional findings, ordering additional imaging or biopsy, image interpretation) and other clinicians (importantly, when ordering imaging studies). As a general effect, these systems should result in an increase in the meaningful and appropriate use of radiology [60]. The available knowledge on medicine and medical imaging is superior to any human ability to memorize and correctly exploit it in favour of patients. Even considering imaging-related articles only, hundreds of new reports appear online daily. When searching PubMed for papers including “imaging” or “radiology”, the number of items obtained per year is 42,757 in 2000, 60,956 in 2005, 94,623 in 2010, 130,353 in 2015; from 2000 to 2015 the number of items per day went up from 117 to 357, more than a three-fold increase [61]. Only the smart use of information technology can allow us for taking advantage of this amount of available information.

Nowadays, when typical randomized controlled trials or large prospective comparative studies imply high costs, proper analysis of the *big data* we already have in our radiology information systems [62] can allow for transforming stored information into new knowledge. In the hierarchy of understanding, an increasing organizational level grows from *data* (discrete elements) to *information* (linked elements), to *knowledge* (organized information), and to *wisdom* (applied knowledge) [63]. Studies on radiological data in this direction are welcome.

#### *Experimental* research in radiology and the structure of the journal

Radiologists will have to contribute to and guide future research projects regarding the above described trends. It is not only a matter of innovation in devices and products, but also a matter of innovation of processes and methods. Testing devices, products and methods in an *experimental* setting is always the first step.

Notably, the word *experimental* has a wide spectrum of meanings, providing many opportunities for this journal. Most commonly regarded as *experimental* are *imaging studies on phantoms, cells, or animal models*. However, we also consider studies in which the observer properly modifies a given practice for a defined outcome to be measured (a planned variation under controlled conditions [64]) as “experimental”. Of course, this applies also to studies on humans and especially on *explorative studies*, such as those reporting secondary end-points of large clinical trials, studies which will also be considered for publication by the journal.

This profile implies a special characteristic of *European Radiology Experimental*: we need a strict cooperation between clinical imaging specialists (radiologists and nuclear physicians) and a large variety of other professionals involved in medical imaging development and application: biologists, chemists, bioengineers and biomathematicians, experts in computer science, information technology and bioinformatics, as well as other physicians working in medical imaging such as pathologists, geneticists, neurologists, surgeons, cardiologists, and many more. The journal will establish a public forum for this large community.

The journal sections are not defined according to the usual organ/system-based or technique-based subspecialties. Open to future changes, we identified eleven fields for submission:

1. Biomathematics and computer modelling
2. Contrast materials, tracers, and theranostics
3. Experimental models of human disease
4. Information technology, big data, image reconstruction and post-processing
5. Imaging biomarkers, radiomics, radiogenomics, and imaging biobanks
6. Interventional
7. Metabolic and functional imaging
8. Methodology and statistics
9. Molecular and hybrid imaging
10. Novel imaging modalities/techniques
11. Radiobiology, radioprotection, and physics in radiology

As per the aims and scope of the journal, about 50% of the Editorial Board members are neither radiologists nor nuclear physicians, thus including expertise from many other fields. I thank all board members for taking part in this initiative.

## Conclusions

Considering the above described trends, *European Radiology Experimental* joins the ESR journal family as an online only and fully (gold) open access journal. This follows the guidelines defined by the *European Union*: papers deriving from projects supported by public funds should be freely available for reading by 2020 [65]. The discussion about free access to data supporting the results of scientific research (the so-called *data sharing* [66]) is ongoing and this accessibility should be combined also with ethical, economic, and authorship issues. *European Radiology Experimental* encourages data sharing and will work in favour of this.

“The future cannot be predicted, but futures can be invented”, said Dennis Gabor, the Hungarian Engineer who received the 1971 Nobel Prize in Physics for the invention of holography [67]. Of course, this cannot be achieved through journals alone, but the ESR is already driving relevant processes, working for the next generation of radiologists. In these changing times, *European Radiology Experimental* can play a pivotal role.
